# Materia Medica, and Therapeutics

**Published:** 1851-01

**Authors:** 


### Part 2—Reviews and Notices of New Works.

### ARTICLE I.

*Materia Medica and Therapeutics, with ample illustrations of Practice in all the departments of Medical Science, and very copious notices of Toxicology, suited to the wants of Medical Students and Practitioners.* By THOMAS D. MITCHELL, A. M., M. D., Professor of the Theory and Practice of Medicine in the Philadelphia College of Medicine; formerly Professor of Chemistry and Pharmacy in the Medical College of Ohio; Professor of Chemistry and Materia Medica in the Medical Department of Transylvania University, Lexington, Ky.; Lecturer on Obstetrics and Diseases of Women and Children; Author of "Elements of Chemical Philosophy," "Hints to Students," etc., etc., etc. Philadelphia, Lippincott Grambo & Co., successor to Grigg Elliot & Co., 14 North Fourth St., 1850, pp., 738, 8 vo. (From the Publishers, for sale by J. Keen Jr. & Brother, Chicago.)

This is a volume containing many excellences, and abounding in blemishes. It is not in our power at this time to review the work thoroughly, but we will endeavor to illustrate the remark with which this article is begun, by a rather cursory reference to Prof. Mitchell's book.

He begins by a long and somewhat rambling introduction, devoted partly to the purpose of a preface, and partly to the *modus operandi* of Medicines. In this he tells us that this volume contains the substance of his lectures as delivered during eleven winters at Lexington. He informs us that he has omitted a large amount of details of the Chemical, Natural, and Botanical history of medicines, supposing it to be better than to swell the volume by adding them. The plan is good, but the defence of it is radically bad. 'It is not because these details are unimportant, that an author is at liberty to omit them, but because they are sufficiently accessible to the profession through other channels; and if we were disposed to be rigidly critical, we might say as much concerning quite a large proportion of the contents of this volume; for if the author had taught us only what was new, we apprehend a small *duodecimo* would have been more to the purpose than a corpulent *octavo*.

We quite agree with him in his belief that the alphabetical arrangement of the articles of the Materia Medica, is much better than any other method of classification, for it would be both burdensome and profitless to treat of a single article under two or more distinct classes. His remarks upon the *modus operandi* of medicines are judicious, and show an independence of hypothesis which is quite noteworthy; but we cannot see that they add greatly to our stock of knowledge on this difficult subject.

The most obvious fault of the book, one indeed which can scarcely fail to be recognized by the most superficial observer, is the inexcusable carelessness with which it is written. Many a lecture will pass muster very well before an audience, which when read will appear in quite another light; and many an anecdote which will set on some quantity of barren spectators to laugh, will be very flat in the perusal. A good lecturer will often prove an indifferent writer, and it requires something more than a good story-teller to convey the point of an anecdote in print. That these remarks are applicable to the work before us, we think no one who will read it can doubt. For instance we find the word *balance* used in the sense of residuum or remainder, and many other vulgarisms equally reprehensible in a standard author; whilst many stale anecdotes are violently lugged in, as it were by the head and shoulders, in the most *mal apropos* manner imaginable.

Another objection that presents itself, is the dogmatic and reckless manner in which the author makes assertions that are to say the least, matters of honest difference of opinion. We doubt whether this is proper even in lecturing before students; but we do know that in the judgment of practical and intelligent physicians, such a method of procedure savors more of the pedagogue than the philosopher.

Passing over the first few pages of the volume, we come to the article *Alcohol*, under which the Professor pauses to consider the Temperance question. After entering his protest against the general use of alcoholic tinctures, he gives utterance to the monstrous assertion that "a large majority of the sots have been made so by the doctors, through the agency of alcoholic medicines." Now we venture to affirm that a more incorrect statement was never couched in bad English. The Professor's remark will doubtless be cited as authority by ranting lecturers, much to the disgust of rational people, and the detriment of the temperance cause. Sweeping assertions of this character, unsustained by any proof whatever, serve no purpose but to prejudice the cause, however good, that the author may advocate. A little further on, the Dr. undertakes to show that alcohol is a poison, although many doubt it. To sustain his position he adduces some curious examples; for instance one man "died in tantly after drinking eight pints of brandy for a wager," and another after drinking a [gallon, we suppose,] bottle of whiskey, and still another had his health seriously injured, by swallowing eight ounces of alcohol, and a boy was nearly destroyed by drinking a pint of gin. Now most intelligent physicians of the present day are agreed, that the habitual use of alcoholic liquors, in moderate quantity, by persons in good health, is much worse than useless; but we apprehend that Dr. Mitchell is the first one to whom it ever occurred, to substantiate the doctrine by citing such terrible illustrations of the fatal effects of enormous quantities of liquor. No man in his senses will doubt that eight pints of brandy drank at one sitting, would kill any man, so, probably, would the consumption of fifty pounds of bacon at a single meal. We are well aware that eight ounces of alcohol at a draught, would produce serious derangement of the digestive and nervous system; but five gallons of milk could not be taken at once without serious inconvenience. So far the argument is as strong against the bacon and milk, as against the alcohol, and this is precisely where the Dr. leaves it. He does not cite either authority or example, which are so abundant, to show that the habitual use of wine, brandy &c., begets diseases of the stomach, liver, and brain, with dropsies, gout, apoplexy, *et cetera*; but simply endeavors to prove that brandy in any quantity must be injurious, because eight pints of it will kill a man; a more obvious *non sequitur* one cannot imagine.

A little further on our author illustrates the moral effect of wine-bibbing, by such apposite illustration as the following:

" *Plutarch's Morals*, published in 1718, at London, has these remarks: 'They usually prove wine-bibbers and drunkards, whose parents begot them when they were drunk; wherefore Diogenes said to a stripling somewhat crack-brained and half-witted, 'surely, young man, thy father begot thee when he was drunk.' ' And Burton, in his celebrated work, called *Anatomy of Melancholy*, remarks, 'If a drunken man gets a child, it will never, likely, have a good brain.' The reasoning connected with these reflections is too copious for our present purpose, but suffices to show that alcohol is the most persuasive of all poisons, and the most ruinous by far, to society.

Now we protest against all such temperance arguments as Prof. Mitchell's and hope that in his next edition he will see fit either to modify or omit them.

One recommendation of the volume is, that it is not burdened as some works on Materia Medica, with a long list of articles, which have ceased to be used by the profession, and another is that the author seems to have well kept in view, his intention to make the work as practicle as possible.

As we observed before, we cannot review the volume, at any great length, but have merely hinted at its prominet merits and defects We have no hesitation in saying that it contains a great deal of useful information, and a rigid revision would secure it a much better reputation than it can ever attain in its present shape. M.

